# Process evaluation of an implementation study in dementia care (EIT-4-BPSD): stakeholder perspectives

**DOI:** 10.1186/s12913-021-07001-2

**Published:** 2021-09-23

**Authors:** Liza Behrens, Marie Boltz, Kiernan Riley, Karen Eshraghi, Barbara Resnick, Elizabeth Galik, Jeanette Ellis, Ann Kolanowski, Kimberly Van Haitsma

**Affiliations:** 1 Ross and Carol Nese College of Nursing, Pennsylvania State University, University Park, PA USA; 2 School of Nursing, University of Maryland, Baltimore, MD USA

**Keywords:** Behavioral and psychological symptoms of dementia, Nursing home, Staff training, Implementation, RE-AIM

## Abstract

**Background:**

Behavioral and psychological symptoms of distress in dementia (BPSD) are major drivers of poor quality of life, caregiver burden, institutionalization, and cost of care in nursing homes. The Evidence Integration Triangle (EIT)-4-BPSD in nursing homes was a pragmatic Hybrid III trial of an implementation strategy to help staff use evidence-based non-pharmacological interventions to prevent and manage BPSD. This study aimed to describe and explore the stakeholders’ perceptions of the process to implement the EIT-4-BPSD strategy including its utility, and the barriers and facilitators to implementation in real-world settings.

**Methods:**

EIT-4-BPSD was a multi-layer implementation strategy that engaged nursing home stakeholder groups to define community specific goals towards reducing BPSD over a 12-month period. Stakeholder groups from nursing homes that completed all 12-months of the implementation strategy were invited to participate in this process evaluation study. Qualitative data from focus group transcripts were analyzed using a conventional content analysis. Emerging codes were sorted into categories, then organized in meaningful clusters based on the domains of the RE-AIM (Reach, Effectiveness, Adoption, Implementation, and Maintenance) framework.

**Results:**

The EIT-4-BPSD implementation strategy was completed in 21 nursing homes; 93 stakeholders participated in focus groups. Over half of participating nursing homes reported meeting their BPSD goals as expected or more. Challenges, facilitators, and contextual factors reported by stakeholder members explains variability in the implementation of EIT-4-BPSD strategy in 11 key categories: family; staff; organizational; staff, environmental, and resident outcomes; utility of EIT resources; adoption barriers and facilitators; care process adaptations; and future planning.

**Conclusion:**

Stakeholders offered guidance on salient factors influencing the feasibility and utility of EIT-4-BPSD adoption and implementation to consider in future implementation research that aims to improve behavioral well-being in NH residents living with dementia. Engagement of family and staff at all levels of the organization (Management, leadership, and direct care); and measurement of staff, environmental, and resident outcomes were perceived as critical for future implementation success. While regulations, finances, and competing demands on staff time were perceived as reducing implementation success.

**Trial Registration:**

The Testing the Implementation of EIT-4-BPSD study was registered in the ClinicalTrials.gov (NCT03014570) January 9, 2017.

**Supplementary Information:**

The online version contains supplementary material available at 10.1186/s12913-021-07001-2.

## Background

There is a glaring need for transparent, pragmatic research in the area of non-pharmacological interventions to manage behavioral and psychological symptoms of distress in dementia (BPSD) [1]. BPSD are conceptualized as behavioral responses to the physical and social environment and include verbal/physical aggression, inappropriate vocalizations, anxiety, psychosis, depression, wandering, resistiveness to care, and sexually inappropriate behaviors [2]. BPSD are not solely related to neuropathology; other social, psychological, and environmental mechanisms underlie their expression [2]. BPSD are major drivers of poor quality of life, caregiver burden, institutionalization, and cost of care [3].

Pharmacological treatments for BPSD have modest effects and are associated with serious adverse events, including death. Alternatively, non-pharmacological interventions are generally safe, exhibiting efficacy when consistently implemented. For this reason, experts recommend use of non-pharmacological interventions as a first-line practice approach despite methodological challenges in testing effectiveness of non-pharmacological interventions [1].

Education alone is not enough for ensuring sustainable practice change; systemic changes are needed. Challenges in creating systemic change in nursing homes (NHs) exist such as: lack of nurse involvement in training and activities, competing intrafacility goals, and high turnover rates of administrators and caregivers [4]. Implementation science frameworks increase effectiveness of adopting evidence-based practice strategies [5] thus, the Implementation of the Evidence Integration Triangle (EIT)-4-BPSD in Nursing Homes was a pragmatic trial using a modified implementation framework to help staff prevent and manage BPSD. A detailed discussion of the EIT framework as used in this trial has been published [6].

Briefly, EIT-4-BPSD is a parsimonious, community-engaged participatory framework of evidence informed approaches to BPSD and a care community stakeholder group, including a site champion, to identify site specific goals for reducing BPSD. Stakeholder groups, composed of individuals within a care community who were committed to changing practices, were responsible for ensuring progress toward goal attainment. Care communities were encouraged to select the following individuals for the stakeholder group: a nurse in a leadership position; a nurse practitioner or physician providing medication management of BPSD; a unit nurse; a nursing assistant; a social worker, an activity staff; a family member, and a resident. Stakeholder groups were assisted by clinical experts (research facilitators), who provided resources for meeting goals and overcoming barriers to successful goal attainment. A designated staff person provided leadership in the day-to-day implementation process (site champion). Consistent with the social cognitive theory [7] the site champion sought to motivate staff to use non-pharmacological approaches for BPSD through role modeling, verbal encouragement, vicarious experiences, and public acknowledgement of successes. The research facilitator met with the champion and stakeholder team for approximately 2 h per month over 12 months. EIT-4-BPSD allows for differences between care communities and encourages tailoring of the implementation process. Table 1 provides an overview of the EIT-4-BPSD implementation strategy and tools. A more detailed summary can be found in Additional file 1.

**Table 1:** Monthly Stakeholder Team Meeting Procedures to Implement EIT-4-BPSD

Activities for Monthly Meetings with Stakeholder Team	EIT-4-BPSD Intervention Month											
	1	2	3	4	5	6	7	8	9	10	11	12
Initial 4-h Brainstorming Session with Site Champion/Stakeholder Team	X											
Step 1: Review of environment and policy assessment		X										
Step 2: Plan and implement education of nurses and families as relevant to the facility goals; plan for new staff education	X											
Step 3: Review Care Plans of consented residents to assure that person-centered approaches to BPSD are established		X	X	X	X	X	X	X	X	X	X	X
Step 4: Review of practical measures and data collected as part of the intervention (e.g., evaluation of care plans; observations of staff during care interactions); Ongoing review of challenges/solutions identified by champion/ stakeholders; review of motivational techniques to assure implementation of person-centered approaches		X	X	X	X	X	X	X	X	X	X	X

*Legend.* During the monthly meeting, the Research Facilitator met with the full Stakeholder Team and worked on the units with the Champion to implement the 4 steps in EIT-4-BPSD (e.g., evaluation and care planning for residents; motivating the staff)

This research aimed to explore stakeholders’ perceptions of the EIT-4-BPSD implementation strategy including its utility, and barriers and facilitators to implementation. We sought to determine what was helpful about the implementation process and what could strengthen successful implementation of the EIT-4-BPSD strategy in the future.

## Methods

### Study design

This process evaluation study used focus groups to describe and understand experiences of NH stakeholders implementing EIT-4-BPSD over 12 months. Focus groups are a widely accepted method to collect qualitative evaluative data in implementation science [8].

### Participants and setting

NHs in Maryland and Pennsylvania were randomized to receive the EIT-4-BPSD approach or standardized education session only for reducing BPSD. Sites who participated in the EIT-4-BPSD arm (*N* = 28) of the study between 2017and 2020 were invited to participate in focus groups. Of the 28 eligible NHs, 21 participated in focus groups between February 2018 and June 2020. Majority of NHs were in urban areas. As shown in Table 2, NHs completing focus groups had a higher average bed size, cared for more residents per day, and experienced higher numbers of health citations in the previous year. Over half (68%) of nursing homes participated in implementation activities as expected or more toward reaching site goals. Reasons for non-participation in focus groups included early withdrawal from the study (*n* = 2), non-allowable entry of research staff related to COVID-19 outbreak (n = 2), and scheduling difficulties (*n* = 3).

**Table 2:** Baseline Population and Setting Characteristics

Characteristic	Did Not Complete Focus Group	Completed Focus Group	Total
**Intervention Homes by State**			
Maryland	5	11	16
Pennsylvania	2	10	12
Total	7	21	28
**Homes that are part of a Continuing Care Retirement Community**			
Total	3	9	12
**Profit Status**			
For Profit	5	10	15
Not for Profit	2	11	13
Total	7	21	28
**Certified Beds**			
Average	113.7	151.1	132.40
Minimum (Maximum)	70 (155)	63 (412)	63 (412)
Standard Deviation	31.9	81.2	73.35
**Residents Perer Day**			
Average	102.6	138.4	120.49
Minimum (Maximum)	60 (144)	55 (351)	55 (351)
Standard Deviation	31.7	75.2	67.98
**Registered Nurse Hours Per Resident Per Day**			
Average	0.7	0.8	0.77
Minimum (Maximum)	.45 (.97)	.38 (1.33)	.38 (1.33)
Standard Deviation	0.2	0.3	0.25
**Licensed Practical NurseHours Per Resident Per Day**			
Average	1.0	0.8	0.87
Minimum (Maximum)	.65 (1.23)	.37 (1.23)	.37 (1.23)
Standard Deviation	0.2	0.2	0.22
**Certified Nursing Assistant Hours Per Resident Per Day**			
Average	2.2	2.3	2.23
Minimum (Maximum)	1.85 (2.4)	1.67 (2.88)	1.67 (2.88)
Standard Deviation	0.2	0.4	0.30
**Total Number of Health Citations**			
Total	51	218	269
Average	7.3	10.4	8.83
Minimum (Maximum)	2 (13)	2 (38)	2 (38)
Standard Deviation	3.5	8.2	7.35
**Federal Fines in the Last 3 Years**			
Total	9	30	39
Average	1.3	1.4	1.36
Minimum (Maximum)	1 (2)	1 (2)	1 (2)
Standard Deviation	0.5	0.5	0.50
**Overall Five- Star Rating Level**			
Average	3.9	3.5	3.67
Minimum (Maximum)	2 (5)	1 (5)	1 (5)
Standard Deviation	0.9	1.3	1.20

*Note.* The “Overall Five Star Rating Level” is based on the Five-Star Quality Rating System implemented in October, 2019 by the US Centers for Medicare & Medicaid Services (https://www.cms.gov/Medicare/Provider-Enrollment-and-Certification/CertificationandComplianc/FSQRS)

All NH stakeholders, including site champion and front-line staff, who participated in the implementation of EIT-4-BPSD activities anytime over the course of the study were invited to participate in a focus group. Because the study’s implementation strategy used a goal-oriented team approach and there was no plan to compare how different types of participants discussed the implementation of the intervention, we opted to interview all stakeholders together despite any potential power differentials between participants [8]. In total, 93 stakeholders participated in focus groups with 1 to 9 stakeholders per group (Table 3). Most were non-Hispanic, Caucasian (74.2%), female (91.4%), and represented various staff roles including: administrators (*n* = 5), directors of nursing (*n* = 17), staff nurses (*n* = 18), nursing assistants (*n* = 6), social workers/social service staff (*n* = 12), recreation therapists/activity staff (n = 17) and others (*n* = 10).

**Table 3:** Demographic Characteristics of Focus Group Participants (*N* = 93)

Participant Characteristics	All Focus Groups		Individual Focus Group (#)																				
	#	%	1	2	3	4	5	6	7	8	9	10	11	12	13	14	15	16	17	18	19	20	21
Gender																							
Male	8	8.6	0	0	0	0	0	0	1	0	0	0	0	1	1	2	0	0	0	0	2	0	1
Female	85	91.4	4	1	6	3	7	8	3	3	4	5	2	3	4	3	5	8	2	2	7	1	4
Race																							
African American	22	23.6	2	0	3	3	0	0	0	0	1	4	1	1	0	2	0	0	0	2	1	0	2
Hispanic	2	2.2	0	0	0	0	0	0	0	0	0	0	0	0	0	0	0	0	1	0	0	0	1
Caucasian	69	74.2	2	1	3	0	7	8	4	3	3	1	1	3	5	3	5	8	1	0	8	1	2
Role in NH																							
Administrator	5	5.3	1	0	0	0	1	0	0	0	0	1	0	0	0	0	1	1	0	0	0	0	0
Director of Nursing	17	18.3	1	0	1	2	2	2	0	1	2	0	0	1	1	1	1	2	0	0	0	0	0
Staff Nurse	25	26.9	1	0	2	0	0	1	2	1	1	3	1	1	1	2	0	2	2	1	2	0	2
Nursing Assistant	6	6.5	0	0	1	1	0	2	1	0	0	0	0	0	1	0	0	0	0	0	0	0	0
Social Worker/Social Service	12	12.9	0	1	0	0	1	1	1	0	0	0	1	0	1	2	0	1	0	0	1	0	2
Recreation Therapist/Activity Staff	17	18.3	1	0	1	0	2	2	0	0	0	1	0	1	0	0	1	1	0	1	4	1	1
Others	10	10.8	0	0	1	0	1	0	0	1	0	0	0	1	1	0	2	1	0	0	2	0	0

### Evaluation framework

RE-AIM [9] was the evaluation framework chosen for this study. RE-AIM includes five dimensions: Reach (the target population), Effectiveness, Adoption (by the setting), Implementation (consistency and adaptations), and Maintenance (of implementation effects in individuals and settings over time). The RE-AIM framework guided formulation of the semi-structured interview guide and data analysis (Table 4).

**Table 4:** RE-AIM Questioning Route for Focus Groups with Intervention Site Stakeholders

Dimension	Example Questions
Reach	You all have been involved in the implementation of EIT-4-BPSD project which began 12 months ago. How would you describe that project to someone who asked-What is that all about?
	What stimulated your participation in this project?
Effectiveness	What changes in care did you notice during the implementation of this project?
	How would you describe factors important to the success of the implementation process? What made the project work?”
Adoption	What stimulated your participation in this project?
	Tell us how this project rolled out in this care community
Implementation	Which were the most important activities you did as part of this project?
	Were any activities not needed or did not produce the results you anticipated?
	What would you describe as barriers to the implementation project?”
Maintenance	Describe what’s happened in your facility since this implementation phase has started?
	What would be the most important aspects to continue forward momentum and sustain the program?”

### Data collection

In-person focus group interviews were led by experienced moderators and lasted approximately 60 min. Two trained note-takers transcribed interview data in real-time. This allowed for transcribed notes to be used immediately as data to be referred to, identification of key concepts as data was being collected, and prompted researchers to closely observe the environment and interactions [10, 11]. The note-takers recorded participant descriptive information, responses to interview questions and any changes in questions or probes on a focus group field notes tool and interview guide. Key concepts discussed during the interview were shared and verified for accuracy with participants. Immediately following focus group sessions, the moderator and note-takers debriefed to review the notes taken by note-takers and document what was learned.

### Analyses

Data from focus groups were analyzed using conventional content analysis [12]. Analytic sessions began after interview guide notes for the first seven NHs were collected and verified for completeness. Three researchers (LB, KR, and MB) independently read the data to familiarize themselves with the content, noting preliminary coding insights. Next, we manually coded significant features of the interview data and developed a standardized codebook. During this phase, several iterative double-coding meetings were conducted until coding agreement was reached. The codebook was shared with the expert investigator team (KV, AK), refined, and then used to code the remaining focus group interview notes. The codebook was iteratively refined as new codes emerged, and subsequently re-validated with investigator team. Coding discrepancies were resolved via consensus. Codes were sorted into categories based on how codes were related and then linked to the dimensions of the RE-AIM framework.

Trustworthiness of data was enhanced by the study team’s methodological expertise and familiarity with NH research; rigorous analytical approaches; use of detailed audit trail; triangulation of data through use of multiple sites; iterative questioning; and confirmation of participant responses during focus group sessions [13].

### Ethical considerations

This study was reviewed by the university institutional review board of the investigators’ two universities and participants provided consent to participate.

## Results

Stakeholders discussed a range of issues influencing the utility and implementation of the EIT-4-BPSD. Table 5 illustrates 11 key categories that emerged from codes organized within in each RE-AIM element.

**Table 5:** Summary of Key Categories Affecting EIT-4-BPSD Program Implementation within RE-AIM Framework

RE-AIM Element	Category	Factors influencing feasibility and utility of EIT-4-BPSD implementation	Exemplar Quote
Reach	Family	Family as essential member of care team	I learn a lot about my residents from families. (3–308)
	Staff	Interdisciplinary team members	Reach out to all of your team members, not just nursing. Everyone can help. (2–306)
	Organizational	Nursing home motivation for study engagement	Although we have many years of experience working with this population, (there were) the failed attempts to get results... (welcomed) a new approach and maybe some education. (3–314)
Effectiveness	Staff Outcomes	Changes in staff behaviors	We also have staff doing things with versus just for residents and we are trying to match activities with resident preferences…nursing assistants are doing more teaching and cueing. (1–108)
		Changes in staff attitudes	We are going towards a prevention mindset to identify and eliminate triggers. It helped them [staff] look at behavior in a different way. (3–122)
		Staff empowerment	Staff have gotten excited about effectiveness of non-pharmacologic interventions, [they] feel empowered. (1–202)
	Environmental Outcome	Changes to the physical environment to promote function and well-being	Definitely focusing more on personal preferences of patients. We have also done things like “soften up the environment.” (1–108)
	Resident Outcome	Decrease in BPSD	Our residents’ behaviors have gone down since the study. (3–314)
Adoption	Utility of EIT Resources	Brainstorming exercise	The brainstorming was really helpful as it helped us to see things in a different way. (2–112)
		DICE model (Describe, Investigate, Create, Evaluate)	They learned to use DICE and used this to figure out the situation and intervene. (2–114)
		Nursing Home Toolkit	Nursing home toolkit website...it was helpful, but we did not utilize too much - enjoyed sundowning tidbit. (2–304)
		Tidbits	We had education sessions using the weekly tidbits, very informal, positive response… (3–310)
		Staff education	I think education is really the most important thing. (2–210)
		Staff contests	The contests were very helpful and got the staff motivated and engaged in providing behavioral interventions to residents. (3–122)
		Behavioral observations	It was a really nice way to give peer-peer observations… first couple of times I found it difficult because I thought I could not intervene, but I learned I could it gave an opportunity to model good responses. It was a real-life example of how to model behavior. (3–310)
		Huddles	…our huddles [are] where we talk about specific residents, their behaviors and what we can do to change their behaviors. (3–314)
		Stakeholder meetings	Stakeholder meeting… we came to monthly meeting open to all staff, much more effective then random sitting next to one another but opportunity for everyone to be equals when approaching this topic. (2–304)
		Interventionist as role model	…really liked…having the research nurse there and doing hands on activities with the staff. (1–108)
		Care plan review	It was very eye-opening…It gave us a chance to look and go through and make sure care plans are accurate. We have written more customized care plans and moved away from check boxes. Extra time to look at and reassess to improve was helpful (3–310)
		Environment and Policy Assessment	…we do have corporate office, and a lot of the policies are handed to the site so you can make suggestions but it is not something you can anticipate a change…so you don’t want to spend time on it. (2–304)
Implementation	Barriers	Physical environment	The environment was a little bit of a barrier for us-no area for open walking. (2–112)
		Nursing home regulations	Worried about HIPPA and getting staff to look at and use this material. (1–106)
		Finances	Many restraints an issue- not able to get finances. (3–308)
		Competing demands	I was promoted which gave me more responsibilities, so I did not have as much time to focus on (implementation) goals. (3–310)
		Staffing levels/turnover	We were working with short staff so a lot of the behaviors they were likely not charting because we were short. (3–314)
	Facilitators	Motivators for change	I think in the beginning we were having a lot of residents with behavioral disturbances and we did not know how to non-pharmacologically manage those residents – that was our largest strive at the time. (2–210)
		Manager/leadership engagement	The manager dedication, house supervisor dedication, and the interventionist following through with what the study offered were all factors to the success of the study. (3–128)
		Staff buy-in	Getting buy-in from staff outside of the stakeholder group. They are all interested, they wanted to learn. The staff genuinely care about our residents. (2–306)
		Access to recreational activities for residents	We made major inroads by establishing that we needed an activities room for the residents. A place that they could go to and engage in some of the activities. We now have that room! (1–106)
	Care Process Adaptations	Staff-staff and leadership communication	Goal number one, we were in the process, but this put an emphasis on ensuring that all staff had access to information. This has made information more accessible. (3–310)
		Care planning process	We started a new behavioral care planning process. We meet weekly to take a deeper dive into why behaviors are occurring, identify and eliminate triggers. (2–306)
		Quantity and quality of resident recreational activities	Cutting down on psychotropics. Increasing activities. We have more resident focused groups now like a men’s group. For the women we had a mother/daughter tea... (2–306)
		Formal process for staff engagement	December in-service was highest attended, very physical in-service where there was competition…they were so engaged…and part of that was that they had to say something important…There was acceptance of time spent… It was worth it. (2–304)
Maintenance	Future Planning	Specific organizational goals	[This study is] an attempt for our organization to improve the well-being of our residents through specific organizational goals. (3–308)
		Remain on Tidbit list	We want to keep getting the tidbits. (2–120)
		Commit to provide in-services	Way to continue in-services…Choose 3 staff to commit to provide in-services. Pick a topic –give open forum to follow-up on the education that received –very positive to continue with all the staff. (2–304)
		Plan for continued meetings	... we still keep the once a month stakeholder meeting. (2–304)
		Measures of success	... we started out thinking about measurement and how to improve our quality indicators, after consulting it seemed that we changed to how we can increase moments of joy as measurement of success (3–308)

[Table 5 should appear about here.]

### Reach

Reach explored characteristics of those engaged in the implementation strategy, including the reasons for enrolling in the study, as compared to those who we intended to engage [9]. Data represented a wide range of stakeholders who engaged in the implementation strategy and reasons for participation in the study from an organizational and individual stakeholder perspective.

Participants described why their NH engaged in the implementation of EIT-4 -BPSD. Some participants described a shared, overt dissatisfaction with the current state of dementia care and a need for more effective approaches.

*Although we have many years of experience working with this population, (there were) the failed attempts to get results...(welcomed) a new approach and maybe some education. (3–314*)

Others described organizational commitments to formalize processes around BPSD assessment and care planning. Desired outcomes included enhanced communication and staff-resident relationships, and improved resident well-being and quality of life. Participants described Administrators’ as key people who advocated for organizational engagement and innovation and were motivated to initiate/sustain academic partnerships to increase resources for care delivery. This motivation was based on past positive experiences and outcomes when working with university research studies. One participant stated:

*All facilities that are invited to participate in dementia research studies should take advantage of it because of receiving extra support, education, and good tips. (2–118)*

Participants described characteristics of EIT-4-BPSD leading to NH engagement including: its participatory nature which was perceived to empower staff (“*give a voice” 3–308)* and align with organizational philosophy of care; and the holistic nature of EIT-4 BPSD, which emphasizes emotional well-being, physical function, and preference congruence.

Participants described staff from various disciplines and departments who participated in implementation activities. They included staff directly recommended by the study protocol such as direct care nursing staff (i.e., nursing assistants, licensed practical nurses, charge nurses, unit managers, nurse educators), unit clerks, activities staff, occupational therapy interns, dietary aides, medical directors, administrator, director of nursing, housekeeping, maintenance, and human resources. Non-nursing team members were described as helpful when cross-trained to tasks that were typically outside of their job. For example, in one NH, nursing assistants lacked time to complete observations of care interactions (EIT-4-BPSD step 4) so they trained unit clerks to complete the observations. Based on experiences like this, participants suggested that staff from all disciplines and shifts be included in the implementation strategy. In contrast, some participants reported that implementation of EIT-4-BPSD was not a priority due to other pressing demands. As one participant reported, *I felt like we had so many problems and that this was another layer (3–314).*

Family members were described as key informants, essential to implementation activities. Participants indicated a need for family education to support non-pharmacological approaches to BPSD. In some NHs family members were involved in implementation activities such as huddles, individual preference-based activities with residents, and education programs. Family member involvement led to better perceived collaboration, communication, and psychosocial support between NH staff and family members. However, in some cases, family was described as creating barriers. For example, in one NH, the stakeholder team identified a need to make environmental changes to include sensory stations in the hallways to promote walking. This change needed family council approval; however, the stakeholder team could not effectively engage the council to discuss this proposal and the initiative failed.

### Effectiveness

Effectiveness explored the positive benefits and negative effects of the implementation strategy [9]. Participants identified positive and negative effects on staff, environmental, and resident outcomes.

#### Staff outcomes

Staff outcomes included changes in staff behaviors and attitudes, and staff empowerment. Participants most often identified increased individualized care for residents as a result of participation in EIT-4-BPSD. Participants discussed avoiding psychoactive medication administration, promoting self-care, providing preference-based activities, and using therapeutic communication including modeling and cueing for residents. Staff were described as doing things “with” residents rather than “for” residents, purposefully matching residents’ preferences with activities to reduce unwanted behaviors and encourage resident well-being. This approach resulted in rippling effects throughout the organization. For example, one participant described:

*…a resident that would never come out of his room. They brought him to the day room and engaged him in music and now he asks for it every day. This has spread through the facility and others ask for music as well. (2–114)*

New staff behaviors were contrasted with past habits of treating behavioral symptoms with medications or ignoring the resident behavior altogether. Participants attributed the changes in staff actions and behaviors to use of the DICE (Describe, Investigate, Create, Evaluate) model (See Table 3).

Participants suggested that changes in staff behaviors resulted from fundamental changes in staff attitudes toward a mindset of identifying and eliminating triggers rather than simply reacting to BPSD or ignoring them altogether. They suggested brainstorming activities, interdisciplinary problem solving, and on-going education and role modeling (by champions and research facilitator) were helpful for staff to reframe residents’ behaviors. This attitudinal change was also associated with a sense of empowerment. As one participant stated:

*Staff have gotten excited about effectiveness of non-pharmacologic interventions, [they] feel empowered. (1–202)*

Empowerment was described as professional growth of nursing assistants and other staff to a place where staff feel valued and competent to contribute, intervene, and even become mentors to others.

Conversely, some participants described discouragement when their ideas were not implemented. For example, one participant rationalized the stakeholder team’s failure to empower staff:

*I think the failure was on the part of the facility not the project itself. When [we] are able to come up with an idea and then make it happen then you are empowered. (2–210)*

#### Environmental changes

Some participants identified changes to the physical environment to promote function and well-being as an implementation outcome, although a few participants did not see the value of environmental modifications. Participants discussed making changes to the physical design and milieu to “soften” the environment and make it more homelike. This included changing lighting, adding new fixtures, playing music, creating activity rooms and/or transforming dayrooms with new paint and activity/life stations for residents based on residents’ past jobs and hobbies and tailored to residents’ current level of function. Some participants expressed frustrations with a lack of progress toward environmental change goals when corporate staff or administrators were not supportive of the change or when there were structural issues that could not be addressed (e.g., long hallways and lack of quiet space).

#### Resident outcomes

Discussed less often, participants perceived a decrease in residents’ BPSD (i.e. calmer, less agitation) resulting from EIT-4-BPSD. This was attributed to resident engagement in activities through life stories and preferences. One participant described a creative way to use a resident’s life story to improve afternoon symptoms of agitation:

*I had a resident who used to have a highball every afternoon. I started giving her some ginger ale …. her afternoon behavior changed/improved. It calmed her right down. (2–114)*

Another participant attributed a general decrease in BPSD among residents to knowing who people are and providing a meaningful and stimulating environment:

*A lot of behaviors in long-term care stem from boredom and lack of stimulation…presence of increased activities…boredom has decreased…improvement in behaviors. (2–204)*

### Adoption

Adoption explored the characteristics of where the implementation strategy was applied and who in the organization applied it [9]. Participants discussed using various resources and tools provided by the study team and their value in promoting uptake of EIT-4-BPSD. With few exceptions, participants described the stakeholder team as the person(s) using EIT-4-BPSD resources within their NH.

#### Brainstorming exercise

Conducted as part of the initial 4-h training, participants described the brainstorming session as a valuable tool to help them think differently about practice issues in their NH. This exercise yielded site-specific implementation goals.

#### Use of the DICE model

Participants reported DICE as a valuable tool to challenge thinking about how to systematically address behaviors. Stakeholders placed information about the DICE model on every unit. Participants also described direct caregivers and social services staff using the model to figure out BPSD situations and intervene.

#### Nursing Home Toolkit Website

The Nursing Home Toolkit [14] was described as a valuable resource for defining behaviors and accessing “Tidbits”. Stakeholders expressed a desire to spend more time looking at the toolkit. Without elaboration, others reported that they did not use it much and it was not “geared toward [Certified Nursing Assistants] CNAs” *(1–202)*.

#### Weekly tidbit emails

Participants frequently reported using the Tidbits as a valuable tool to educate and motivate staff (*very informal…. positive response, 3–310)*. Tidbits were printed, put into notebooks, and posted on units to encourage staff to refer to them. Participants described value in providing ongoing education about BPSD when orienting new staff and in routine in-servicing of existing employees.

#### Staff education

Participants suggested that education around BPSD helped staff learn tips and techniques to address behavioral situations with residents. Participants found that this was particularly helpful when it was conducted face-to-face with hands on exercises and role plays and included staff from all departments and disciplines in the NH.

#### Staff contests

Contests were described as valuable ways to engage staff in providing BPSD interventions for residents. They also raised awareness of BPSD and promoted shared ideas to support resident well-being.

#### Behavioral observations

Observations of staff-resident interactions were conducted by stakeholder team members, organizational leaders and management, staff peers, and unit clerks. Participants described pragmatic behavioral observations as a valuable way to provide feedback to staff, identify staff that were appropriate for dementia care units, track occurrence of behaviors, and evaluate progress toward reaching study goals to reduce BPSD. Some participants felt the observational tool was not user friendly.

#### Huddles

Huddles, brief “stand-up’ meetings, were described as a valuable, efficient tool to discuss specific residents, their BPSD, life histories and preferences, and what the team can do to promote resident well-being. Some sites conducted huddles weekly and others monthly.

#### Stakeholder meetings

Participants described monthly stakeholder meetings as a valuable space for consulting and following-up on a goal or approach to make the change a reality. The site champion would organize the stakeholder meeting and attempt to include representatives from all departments. In some cases, lack of administrator engagement and time constraints made it difficult for the champion to organize stakeholder meetings, and some stakeholders suggested that they did not see any benefit from them at all.

#### Interventionist as role models

Participants described working with the research facilitator to provide: hands-on activities and education to staff and site champions; “fresh eyes” in challenging BPSD situations; a listening ear and continual encouragement to team members; a structure for team members to track tasks toward goal attainment; an education with family members. Participants valued the research facilitators, often suggesting they would have liked more *“face to face time” (1–108)* with them. Stakeholders acknowledged the Environment and Policy Assessment, conducted by the research facilitator and champion, but did not describe it as integral to implementation.

#### Care plan review

Stakeholders discussed reviewing care plans as a valuable way to individualize care. They suggested care plan audits-review of care plans using a study provided checklist-were useful for evaluating completeness and enhancing person-centered approaches.

### Implementation

Implementation explored how consistently EIT-4-BPSD was delivered, how it was adapted [9], and barriers and facilitators to implementation [15]. Three categories emerged: barriers, facilitators, and care process changes.

#### Barriers

Participants described barriers related to implementing and adhering to all EIT-4-BPSD protocol components. These included the physical environment, NH regulations, finances, competing demands, and staffing levels/turnover. Lack of physical space impeded stakeholder efforts to make the environment more person-centered, i.e., homelike, and reflective of the residents’ preferences and backgrounds. Regulations curtailed the sharing of personal resident information among staff and union contracts prohibited peer-peer behavioral observations, which were designed to provide feedback on the implementation of the care plan. Participants described finances as a barrier when they were unable to secure funding for organizational strategies. For example, one participant described the stakeholder teams’ goal to add signage within the unit to facilitate wayfinding, which was not met due to an inability to secure funding.

Participants often reported competing demands on staff time as a barrier to implementation. In these cases, the approach to care was described as time focused and task oriented and staff were described as stressed and overwhelmed with trying to balance BPSD approaches (e.g. adapted communication and therapeutic activities) with meeting the residents’ personal care needs. Implementation activities were viewed as “extra” activities that staff had to balance with job demands. For some, clinical issues and surveys took precedence over participation in implementation activities (e.g., stakeholder meetings and education). One participant described demands associated with changing levels of responsibilities detracting from time needed to work on implementation goals:

*I was promoted which gave me more responsibilities, so I did not have as much time to focus on (implementation) goals. (3–310)*

Also frequently discussed were issues in staffing levels and turnover. As one.

participant reported:

*There were challenges due to turnover in key staff, plus the day to day struggle of juggling priorities. (2–306)*

Participants from NHs that identified problems with staffing levels also described turnover in administrative, activities, social work, and direct-care staff occurring as frequently as every 3 months. Some reported using a high volume of agency staff and regular staff working double shifts resulting in worker fatigue and burnout. This made it difficult to hold staff accountable to maintain care consistency. It also required stakeholder teams to continually repeat training on BPSD and at times required a change in site champion. Based on perceived poor implementation, participants recommended that NHs *“get the right champion from the beginning” (2–210)*. Participants explicated ideal characteristics of successful champions as people who are strong, passionate about dementia care, and able to make change.

Participants opined that staff turnover was a good thing, by removing staff that “*just don’t make the cut*” *(1–104).* One participant described a decrease in BPSD resulting from removing staff that were unsuitable for the memory care unit. Another described turnover offering new opportunities for other leaders to emerge and take-on responsibilities to disseminate the intervention. This was illustrated by one participant, describing the administrator’s employment termination: *Once we lost the administrator piece, it gave us more responsibility to get the word out. (1–206).* Conversely, participants who described positive staffing levels with tenured administrators, also reported that the program was achievable due to fewer competing demands on staff time and less staff burnout.

#### Facilitators

Discussed more frequently than barriers, participants described several workforce characteristics promoting consistent adherence to EIT-4-BPSD. Implementation facilitators included motivators for change, manager/leadership engagement, staff buy-in, and access to recreational activities. Participants discussed motivators for change as reasons why it was important to participate in the study and implement the intervention strategies. These varied between staff and leadership. Staff were described as having a desire to learn behavioral approaches to reduce negative effects of living with dementia for residents and thus provide quality dementia care and regulatory compliance. One stakeholder described this as:

*I think in the beginning we were having a lot of residents with behavioral disturbances and we did not know how to non-pharmacologically manage those residents – that was our largest strive at the time. This was resulting in mandatory reporting to the state. (2–210)*

Closely related, participants expressed staff motivations to improve the residents’ quality of life.

*So many opportunities to learn new and different ways to work with residents…this was real-life and real-time…we are coming to the table with specific issues and leaving with the highest quality of life. (2–304)*

Participants described managers as the driving force behind consistent participation and follow through with implementation activities. Participants described engaged managers as people who were dedicated and willing to improve dementia care:

*The manager dedication, house supervisor dedication, and the interventionist following through with what the study offered were all factors to the success of the study. (3–128)*

Engaged leaders provided encouragement and support to staff with material resources and decision-making and stayed fully engaged in implementation activities (e.g. stakeholder meetings). Participants also indicated staff buy-in was critical to implementation success. Staff buy-in was described as the staffs’ acceptance and support of implementation activities and willingness to try new things to manage BPSD.

Some participants suggested that having access to a dedicated space and supplies for activities facilitated consistent implementation efforts. Having activities readily available helped engage residents as discussed earlier and supported strengthening staff-resident relationships.

#### Care process changes

Participants discussed making process changes in care delivery to incorporate implementation activities into routine operations. In response to difficulties in getting information passed on to staff about residents and implementation activities, as well as shift to shift blaming for lack of follow-through, some NHs focused on improving processes around staff-staff and leadership communication. One participant stated:

*Goal number one, we were in the process, but this put an emphasis on ensuring that all staff had access to information. This has made information more accessible. (3–310)*

Important information to communicate among staff included specific information on residents’ behaviors and needs, and individualized approaches. The most common process changes employed to improve communication was the addition of weekly one-on-one meetings with staff and/or weekly team huddles to discuss specific residents experiencing BPSD and adding the research facilitator (as a behavioral expert) to mood and behavior rounds. One NH reported scheduling huddles immediately after weekly care conferences to support consistency. Participants from these homes discussed the importance of creating a non-threatening, constructive social environment where staff were open to new ideas, felt comfortable asking questions about things they did not understand, and provided regular feedback and dialogue about residents.

Some stakeholders described adapting care planning processes to promote implementation of EIT-4-BPSD:

*We started a new behavioral care planning process. We meet weekly to take a deeper dive into why behaviors are occurring, identify and eliminate triggers. (2–306)*

NHs who made process changes to implement EIT-4-BPSD described moving away from check-box care plans provided by electronic medical records to more customized individual care plans written free hand. For example, one participant described an organization-wide documentation program that included residents’ behaviors, triggers, and interventions in their electronic medical record system where nurses are required documents. They also described changing verb tense of care plans to first person, called “I” care plans. Other participants described creating processes to share individualized care plan information with front-line staff by either creating a resident summary or adding information to existing tools used by direct-care staff. Not all NHs implemented changes in care planning processes; some expressed that their care plans were already person-centered, and changes were unnecessary.

A few participants reported making process changes to improve the quantity and quality of recreational activities offered to residents, and by extension family members. Meaningful activities that were engaging, resident focused [based in resident preferences], and addressed BPSD in place of medications. This included music and memory programs, and gender specific programs. One stakeholder illustrated the importance of meaningful activities:

*Cutting down on psychotropics. Increasing activities. We have more resident focused groups now like a men’s group. For the women we had a mother/daughter tea... (2–306)*

Staff engagement to provide residents with activities was essential, especially within the activities department and CNA staff. Participants from one NH described making significant changes to the activity department structure; they now hire people with specific skills to implement projects. Another NH started family education nights to facilitate family engagement in interventions to reduce BPSD.

Directly addressing challenges in staff engagement, some NHs created formal processes to engage staff in performance improvement activities around BPSD. For example, one participant described successful changes to behavioral rounds procedures including moving the time and location of meetings to better engage front-line staff and sending a clear invitation to the meeting with times and expectations for participation. Once invited, engaging staff on regular basis with “high energy”, facilitators provided motivation for continued participation.

### Maintenance

Maintenance explored the sustainability of the EIT-4-BPSD program as part of the regular routine of the organization [9]. Predominantly describing organizational level commitments to continue implementation activities, one distinct category of future plans for implementation emerged. Participants from over half of the NHs described a desire to continue to develop and advance specific organizational goals, continue weekly behavioral meetings, remain on tidbit lists, provide educational in-services on BPSD to staff, and monitor measures of success.

## Discussion

This multi-site study explored the views of various stakeholders regarding EIT-4-BPSD implementation in NHs. We identified challenges, facilitators, and contextual factors that explain variability in the implementation of EIT-4-BPSD across sites and presented them within the RE-AIM framework. As such, this study contributes to the recognized need to evaluate evidence-based protocols for BPSD [2]. The reach of site level engagement in the study represented a readiness to adopt evidence based BPSD approaches. Organizational and stakeholder reasons for participating were aligned with Holt and colleagues’ [16] conceptualization of organizational readiness: perceived appropriateness (descriptions of philosophical alignment and benefits to the resident); managerial support (e.g., administrator as key to implementation); self-efficacy (i.e., desire for improved knowledge and skills); and personal valence (the belief that engagement would empower staff). When EIT-4-BPSD was implemented, it appeared to reach across departments and staff roles, and was inclusive, as there were no descriptions of residents with BPSD who were deemed to be inappropriate and excluded.

Family and residents were suggested by the protocol as essential stakeholder group members; however, participants only described the essential contribution of families to EIT-4-BPSD implementation. Their contribution to resident well-being is corroborated by research that demonstrates family involvement in information sharing, planning, and evaluating care, and providing psychosocial support promotes quality of life [17, 18]. However, despite participants’ reports of family engagement in EIT-4-BPSD as recipients of education and as partners in individual residents’ care delivery, families were not represented as stakeholders. Future implementation research should consider approaches to engage families and residents as partners in the planning and evaluating the systemic implementation of EIT-4-BPSD.

As described by the participants, the benefits of EIT-4-BPSD are noteworthy for their breadth (staff, environmental, and resident outcomes), and the potential insights into what staff and stakeholders perceive as meaningful and relevant measures to be considered in NH implementation research. They described, in detail, more positive staff behaviors and attitudes. They associated these changes with less BPSD in residents as well as staff feelings of empowerment, wherein staff can effect positive change. These findings point to the critical importance of including staff outcomes in pragmatic implementation trials.

The most problematic BPSD discussed by stakeholders were agitation and aggression. Stakeholders associated boredom with BPSD. However, there was no mention of apathy or depression, which are common in NH residents with dementia; and are more likely to go undetected and thus under-managed, increasing the risk for diminished quality of life and function, and increased morbidity and mortality [19, 20]. It is possible that staff use “boredom” to describe the lack of interest, initiative, and indifference to activities in the environment commonly associated with symptoms of apathy and passivity in dementia [21]. Additionally, quantitative results of the EIT-4-BPSD study indicated no significant effects for reducing depressive symptoms, agitation, or resistiveness to care [22] further suggesting that future work needs to evaluate staff detection and understanding of all BPSD.

Not uncommon, stakeholders viewed adoption of the behavioral approaches to BPSD as a low priority as compared to competing organizational demands [4]. Conversely, stakeholders’ expressed motivation to improve the residents’ quality of life often spurred adoption of EIT-4-BPSD. Thus, a collective appraisal of leadership and staff attitudes towards adoption of evidence-based behavioral wellness programs might be an important pre-implementation activity. The Evidence-based Practice Attitude Scale [23] is a pragmatic measure of eight dimensions of attitudes towards adoption of evidence-based practices (EBP) such as intuitive appeal of EBP, likelihood of adopting the EBP, openness to new practices and perceived divergence from usual practice, that can be used in future research.

Described as critical, the role of committed unit managers on the stakeholder team was important for the integration of EIT-4-BPSD into the residents’ care and daily operations. This finding is consistent with research that suggests unit managers as critical change agents who support adoption of practice change by diffusing and synthesizing information and integrating the innovation into daily practice while “selling” the implementation [24]. Also consistent with previous implementation research [25, 26], the stakeholders offered clear direction about the resources that supported adoption of EIT-4-BPSD, and include all four steps of the strategy (staff education; care planning processes; staff mentoring, and attention to promoting an environment that supports function, social engagement, and comfort).

Not surprisingly, low staffing numbers were stated as a barrier to the implementation of EIT-4-BPSD; however little discussion focused on the deployment of staff (i.e., what staff are actually doing and when). Participants described the need to prioritize personal care tasks (e.g. bathing) over psychosocial interventions, suggesting a dichotomization of resident needs that is not conducive to person-centered care. Person-centered dementia care emphasizes meeting emotional, social, and practical needs, an approach essential to promoting a sense of well-being in persons with dementia [14]. Findings suggest the need for time studies that evaluate staffing hours necessary to provide individualized care to persons with dementia, in order to establish staffing patterns that align with high quality, person-centered care including EIT-4-BPSD.

The environment was perceived as an important consideration when implementing EIT-4-BPSD, including its capacity to support meaningful activities and a familiar, homelike setting. An investigator -developed tool to examine the safety, functionality, and comfort of the NH environment was used to implement EIT-4-BPSD [27]; however, the participants did not describe its use. This is an area for future research, including the alignment with resident preferences and health care goals. In this study, a few participants refer to contextual factors as barriers to implementation. They briefly describe constraints posed by financial/reimbursement status, staffing levels, and regulatory demands, all areas worthy of deeper and broader examination in NH implementation research.

The relatively short implementation timeframe of this study did not allow for a robust examination of maintenance of the EIT-4-BPSD strategy. There were signs that systems were in place to support sustainability, such as routine huddles and integration of BPSD in ongoing staff education and quality assessment/improvement activity.

### Implications

Findings from this study offer guidance on salient factors to consider in future implementation research aiming to improve behavioral well-being in NH residents with dementia. First, a pre-appraisal of organizational readiness, as recommended by Weiner [28] would support organizational engagement and customized implementation strategies. The use of an implementation framework that includes known constructs of readiness such as leadership engagement, knowledge access, and resource availability would better capture the factors influencing implementation. This appraisal should include an alignment of organizational philosophy with the premise of the innovation. Additionally, unit nurse managers, although historically underrepresented in research [24], are key to the implementation of new interventions. Finally, staff outcomes, such as behavior change and perceptions of empowerment, are important considerations when developing implementation goals.

### Strengths and limitations

Our analysis included data from 21 sites that were diverse in quality star rating criteria and profit status but were not diverse in terms of rurality. We opted to conduct one focus group per research site potentially skewing results based on differences in expertise in BPSD management between supervisors and direct care staff and power differentials for enacting practice changes within groups. Study participants were representative of typical NH staff; however, we were unsuccessful engaging residents and families in focus groups, thus not able to effectively represent their views on implementation efforts and potentially skewing results in favor of the implementation strategy. Additionally, we did not use audio recording potentiating loss of information and valuable details. Despite these limitations, our rigorous notetaking and content analysis procedures by an expert team of qualitative researchers facilitated data depth and trustworthiness [11, 12].

## Conclusions

The EIT-4-BPSD is a multi-layer, integrated set of implementation strategies geared to promote success of NH stakeholders to adopt evidence-based approaches to prevent and manage BPSD experienced by NH residents. While our quantitative data did not show efficacy in resident outcomes [22], we identified multiple factors influencing feasibility and utility of EIT-4-BPSD adoption and implementation in NHs. Overall, based on stakeholder report, the EIT-4-BPSD strategy as a process, can be successfully implemented in NHs and helpful in changing staff’s approach to BPSD.

## Supplementary Information

**Additional file 1.**

## Data Availability

Data can be made available by reasonable request to the corresponding author.

## Abbreviations

BPSD: Behavioral and Psychological Symptoms of Distress in Dementia
CNA: Certified Nursing Assistant
COVID-19: SARS-COV-2 virus
DICE: Describe, Investigate, Create, Evaluate
EBP: Evidence Based Practice
EIT: Evidence Integration Triangle
EIT-4-BPSD: Evidence Integration Triangle for Behavioral and Psychological Symptoms of Distress in Dementia
NH: Nursing Home
RE-AIM: Reach, Effectiveness, Adoption, Implementation, and Maintenance
