# Supplementary material for: Process evaluation of an implementation study in dementia care (EIT-4-BPSD): stakeholder perspectives
Source: BMC Health Serv Res. 2021 Sep 23;21:1006. doi: 10.1186/s12913-021-07001-2 (PMC8458006; doi:10.1186/s12913-021-07001-2)
Supplement: Supplementary file 1 — Additional file 1. [file 12913_2021_7001_MOESM1_ESM.docx]

**Additional File 1**

**Implementation Framework**

The EIT-4-BPSD implementation strategy incorporates evidence-based approaches to promote well-being in NH residents living with dementia, with the Evidence Integration Triangle (EIT) implementation framework. EIT contains three elements: participatory implementation process with a recommended group of stakeholders and a site champion; implementation of evidence‐based approaches; and practical progress measures. Quantitative measures of progress were practical and known to be meaningful, and included organizational outcomes (changes in the environment, policies, and care plans) and resident outcomes (use of medication for behavior management, physical function, mood, quality of life, anxiety, resistance to care, falls, and hospitalizations).

The EIT-4-BPSD implementation strategy is led by a research facilitator with advanced healthcare education and experience in long-term and dementia care, empowers NH stakeholders to develop and implement customized, organizational strategies for person centered care. The stakeholder team was composed of committed members who assist with organization-side changes needed to appropriately manage BPSD. The recommended facility stakeholder team included: a nurse in a leadership position (e.g., director of nursing); a unit nurse; a nursing assistant; a family member; an activity staff; a social worker; a resident; and any other team member per facility preference. At the initial stakeholder meeting, the stakeholders identified unique barriers to person-centered care and potential ways to address those barriers with best practices, establishing goals to be met over the year-long implementation period.

In addition to the stakeholder team, a research facilitator worked with a site champion-an employee of the targeted NH community-who helped support implementation of EIT-4-BPSD. The research facilitator, champion, and stakeholder team met monthly for 12 months to implement the four steps of the EIT-4-BPSD procedures: 1) assessment of the environment and policies; 2) staff education; 3) establishing person-centered , evidence-informed plans; and 4) ongoing mentoring and motivating staff. The research facilitator was available via phone or email during regular business hours for individual coaching sessions with champions. All sites received weekly “tidbit” emails which provided booster education and innovative strategies for behavioral approaches to BPSD. Motivational strategies, such as contests, were used to promote engagement of stakeholders in intervention procedures.
